# Cohort Profile: East London Genes & Health (ELGH), a community-based population genomics and health study in British Bangladeshi and British Pakistani people

**DOI:** 10.1093/ije/dyz174

**Published:** 2019-08-28

**Authors:** Sarah Finer, Hilary C Martin, Ahsan Khan, Karen A Hunt, Beverley MacLaughlin, Zaheer Ahmed, Richard Ashcroft, Ceri Durham, Daniel G MacArthur, Mark I McCarthy, John Robson, Bhavi Trivedi, Chris Griffiths, John Wright, Richard C Trembath, David A van Heel

**Affiliations:** 1 Blizard Institute, Barts and the London School of Medicine and Dentistry, Queen Mary University of London, London, UK; 2 Wellcome Trust Sanger Institute, Wellcome Trust Genome Campus, Hinxton, UK; 3 London Borough of Waltham Forest, Waltham Forest Town Hall, Walthamstow, UK; 4 Department of Law, Queen Mary University of London, London, UK; 5 Social Action for Health, London, UK; 6 Analytic and Translational Genetics Unit, Massachusetts General Hospital, Boston, MA, USA; 7 Program in Medical and Population Genetics, Broad Institute of MIT and Harvard, Cambridge, MA, USA; 8 Wellcome Centre for Human Genetics, University of Oxford, Oxford, UK; 9 Oxford Centre for Diabetes, Endocrinology and Metabolism, University of Oxford, Churchill Hospital, Oxford, UK; 10 Oxford NIHR Biomedical Research Centre, Churchill Hospital, Oxford, UK; 11 Bradford Institute for Health Research, Bradford Teaching Hospitals National Health Service (NHS) Foundation Trust, Bradford, UK; 12 School of Basic and Medical Biosciences, Faculty of Life Sciences and Medicine, King’s College London, London, UK

## Why was the cohort set up?

East London Genes & Health (ELGH) is a community based, long-term study of health and disease in British Bangladeshi and British Pakistani people in east London. ELGH has a population-based design incorporating cutting edge genomics with electronic health record (EHR) data linkage and targeted recall-by-genotype (RbG) studies. ELGH currently has 38 899 volunteers and is actively recruiting with funding to expand to 100 000 volunteers by 2023. ELGH is an open access data resource, and its research will impact upon a population at high need and will redress the poor representation of non-White ethnic groups in existing population genomic cohorts.^1^

Almost a quarter of the world’s population, and 5% of the UK population, are of South Asian origin.^2^ The risk of coronary heart disease is three to four times higher, and of type 2 diabetes (T2D) two to four times higher, in British South Asians compared with White British people.^3^^,^^4^ East London incorporates one of the UK’s largest South Asian communities (29% of 1.95 million people), of which 70% are British Bangladeshi and British Pakistani, and its population live in high levels of deprivation (Tower Hamlets, Hackney, Barking and Dagenham are the 9th, 10th and 11th most deprived local authorities in England).^5^ Compared with White British people, British South Asians living in east London have a 2-fold greater risk of developing T2D,^6^ nearly double the risk of non-alcoholic liver disease^7^(many volunteers are practising Muslims and do not drink alcohol) and over double the risk of multimorbidity,^8^ with the onset of cardiovascular disease occurring 8 years earlier in men.^8^ Determinants of poor cardiometabolic health start early in the life course, and east London rates of overweight and obese children are among the highest in the UK.

Recent genomic advances offer exciting potential to better understand the genetic causation of disease,^9^ including rare loss-of-function gene variants.^10^ Genetic variation relevant to British Bangladeshi and British Pakistani populations, such as autozygosity arising from parental relatedness, is under-researched with regards to potential effects on complex adult phenotypes at a population level.^11^^,^^12^

ELGH fosters authentic, inclusive, long-term engagement in its research, to deliver future health benefits to the population it represents. Community involvement in ELGH helps prioritize areas for research, including T2D, cardiovascular disease, dementia and mental health. ELGH undertakes a range of public engagement work, including collaboration with the award-winning Centre of the Cell.^13^

## Who is in the cohort?

**Figure 1.**
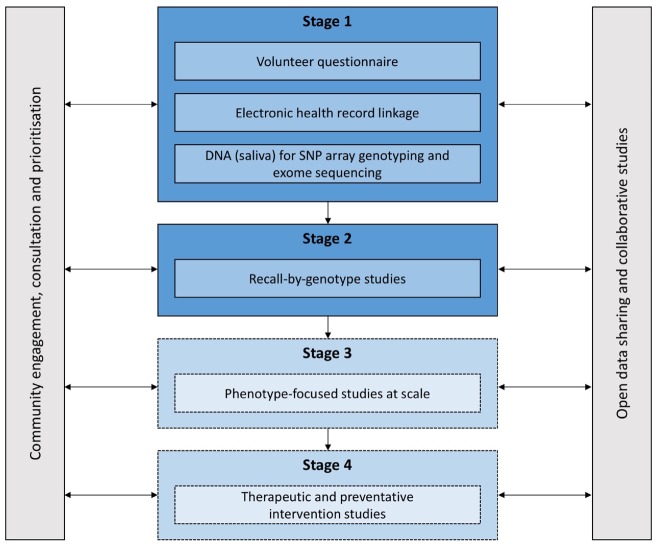
ELGH study design. Stage 1 and 2 studies have commenced. Stage 3 and 4 studies will commence in 2019.

ELGH (see Figure 1) incorporates population-wide recruitment to Stage 1 studies, and targeted recruitment to Stage 2 recall-by-genotype (RbG) studies. Stage 3 and 4 studies are planned.

During Stage 1, ELGH invites voluntary participation of all British Bangladeshi and British Pakistani individuals aged 16 and over, living in, working in or within reach of east London. Recruitment is largely undertaken by bilingual health researchers, and takes place in: (i) community settings, e.g. mosques, markets and libraries, supported by a third-sector partner organization (Social Action for Health); and (ii) health care settings, e.g. GP surgeries, outpatient clinics. Stage 1 volunteers complete a brief questionnaire, give consent to lifelong EHR linkage and donate a saliva sample for DNA extraction and genetic tests. Between April 2015 and mid-June 2019, ELGH recruited 38 899 volunteers to Stage 1. At the most recent data linkage (November 2018), 97% of 31 646 had valid NHS numbers: 61% had linked primary care health record data available; 84% had linked secondary care data. By 2020, near-complete (>95%) linkage to primary care health records is expected with improved data connectivity, supported by Health Data Research UK. Recruitment into outer London regions, and a new study site in Bradford, are planned for 2019/20, areas with similar ethnic populations and comparable health needs.

**Figure 2.**
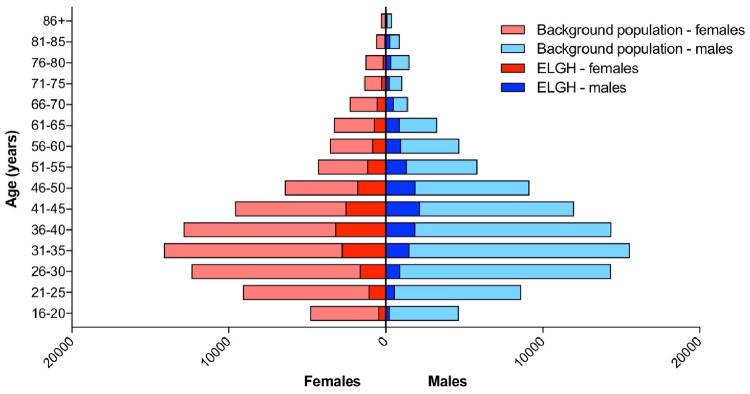
Population pyramid showing age and sex of ELGH volunteers (*n* = 29 370) versus the total population of British Bangladeshi and British Pakistani people (*n* = 152 564) in east London (all NHS GP-registered adults residing in the London Boroughs of City and Hackney, Newham, Tower Hamlets, Waltham Forest), aged ≥ 16 years.

Summary data from the Stage 1 volunteer questionnaire and EHR data linkage are presented in Table 1, including both baseline and longitudinal health data. Basic demographics of ELGH volunteers are compared with population-wide data in Figure 2, and highlight that the convenience sampling approach in Stage 1 recruitment has achieved a sample broadly representative of the background population with regard to age and sex, but which modestly favours recruitment of women over men in those aged <45 years. ELGH volunteers live in areas of high deprivation (97% in the most deprived two quintiles of the Index of Multiple Deprivation). Parental relatedness is reported by 19% of ELGH volunteers.

**Table 1.** Baseline characteristics of ELGH volunteers from self-reported questionnaire and electronic health record data

**Self-reported questionnaire data (*n* = 31 646)**	
Year of birth *n* = 31 634	Median 1977
	Interquartile range 1967-85; range 1915-2002
Sex *n* = 31 639	Male *n* = 13 928 (44%)
	Female *n* = 17 710 (56%)
Ethnicity *n* = 31 508	Bangladeshi or British Bangladeshi *n* = 21 083 (67%)
	Pakistani or British Pakistani *n* = 10 319 (33%)
Parental relatedness	Yes = 5959 (18.8%)
	No = 24 601 (77.8%)
	Don't know = 1013 (3.2%)
	Not documented = 77 (0.2%)
**Linked electronic health record data (*n* = 21 514)**	
Index of Multiple Deprivation (2015)	Quintile 1 (most deprived), *n* = 12 551 (58%)
	Quintile 2, *n* = 8400 (39%)
	Quintile 3, *n* = 411 (2%)
	Quintile 4, *n* = 131 (0.6%)
	Quintile 5 (least deprived), *n* = 11 (0%)
Number of ELGH volunteers with common conditions:	
Type 2 diabetes	4769 (22%)
Hypertension	3956 (18%)
Ischaemic heart disease	1048 (4.9%)
Dementia	47 (0.2%)
Asthma	2297 (10.7%)
COPD	255 (1.2%)
Number of ELGH volunteers with commonly recorded clinical data measured in the past 5 years:	
Body mass index	18 654 (86.7%)
	Mean = 27.16kg/m^2^
	Standard deviation = 4.87kg/m^2^
	Range = 14–69kg/m^2^
Smoking status	18 938 (88%)
	Never smoked = 14 353 (76%)
	Ex-smoker = 2146 (11%)
	Current smoker = 2276 (12%)
	Uninformative coding = 194 (1%)

ELGH operates under ethical approval, 14/LO/1240, from London South East NRES Committee of the Health Research Authority, dated 16 September 2014.

## How often have they been followed up?

ELGH contains real-world EHR data, its collection triggered by a broad range of clinical encounters including routine and emergency care. East London has an extensive track record of using routine clinical health care data (predominantly from primary care) in research studies.^6^^,^^7^^,^^14^ Electronic performance dashboards are embedded in clinical practice, facilitating high quality and equitable disease screening and clinical care.^15^^,^^16^ Primary care health records were digitized around 2000 and offer a rich source of data on clinical encounters since then, but also include pre-digitization dates of diagnoses and summarized clinical events (e.g. type 2 diabetes, diagnosed in 1992). Health data linkage and extraction takes place 3-monthly and ELGH volunteers have consented for lifelong EHR access, facilitating longitudinal follow-up.

ELGH can invite volunteers to Stage 2 studies up to four times per year for more detailed study visits, e.g. recall by genotype (RbG) and/or phenotype, for clinical assessment and collection of biological samples, subject to ethics approval, volunteer acceptability and community advisory group approval. As at August 2019, around 60 ELGH volunteers have participated in Stage 2 RbG studies.

## What has been measured?

Available data are summarized in Table 2.

**Table 2.** Summary of all data types currently available in ELGH for Stage 1 volunteers, and planned for late 2019 onwards

Data source	Data fields	Volunteers	Duration of data collection
Volunteer questionnaire	Basic details: name, date of birth, ethnicity, address, GP, NHS number	All	Cross-sectional at study entry
	Self-reported diabetes status		
	Self-reported parental relatedness		
	Self-assessment of overall health and well-being		
Electronic health records	Primary care (GP) records (currently coded in READ2 or CTV3 clinical terminologies):	All volunteers where data linkage is possible (currently 61%, due to increase to >95% in 2019 with linkage to a wider GP practice network)	Real-world data with access to all available historical (retrospective) data and lifelong (prospective) data
	Sociodemographic data		
	Diagnoses		
	Prescribing data		
	Clinical measurements, e.g. height, weight, body mass index, blood pressure		
	Laboratory tests (e.g. blood tests)		
	Care processes, e.g. referrals		
	Quality and Outcomes Framework indicators: including care processes and outcomes for common diseases (e.g. diabetes, asthma, depression), public health concerns (smoking, obesity) and preventative measures (e.g. blood pressure checks)		
	NHS Health Checks: screening for diabetes, heart disease, kidney disease, stroke and dementia offered to 40–74 year olds		
	Secondary care (hospital) records:	All volunteers in contact with secondary (hospital) care	Real-world data, includes retrospective data since 2012 and lifelong (prospective) data
	Diagnoses (ICD10) and procedures (OPCS-4)		
	Chronic problem listing (SNOMED)		
	Laboratory tests (e.g. blood tests, histopathology, microbiology)		
	Maternity records		
External health record data sets and registries	Hospital Episode Statistics	All volunteers (planned)	Real-world data, includes retrospective data since 2003, and lifelong (prospective) data
	Office for National Statistics mortality data		
	National Cancer Registration and Analysis Service, NCRAS		
	National Cardiovascular Outcomes Research: national audits		
Genetic investigations	Whole exome sequencing	All volunteers reporting parental relatedness (currently 19%)	Not applicable
	Illumina Infinium Global Screening Array v3.0+MD	All volunteers up to 50 000 (to be undertaken in early 2019)	Not applicable
Recall studies	Bespoke clinical phenotyping and sample collection according to genotype of interest. Core samples taken on all for methylation assays, transcriptomics, lipidomics and metabolomics	60 volunteers to date, with approval for all volunteers to be approached for recall up to four times per year	Dependent on protocol

- **Volunteer questionnaire** (Supplementary File 1, available as Supplementary data at *IJE* online). This self-report questionnaire collects brief data including: name, date of birth, sex, ethnicity, contact details, diabetes status, parental relatedness and overall assessment of general health and well-being. The questionnaire has been designed to facilitate high throughput recruitment and volunteer inclusivity where language and cultural differences may exist, and to be used with or without researcher assistance. The questionnaire does not capture environmental factors (e.g. no self-reported data on smoking, alcohol, diet, physical activity—although smoking and alcohol use are available from other data sources, discussed below). Completion of the volunteer questionnaire triggers health record data linkage via NHS number.
- **NHS primary care health record data linkage.** We first design data extraction in human-readable form (Supplementary File 2, available as Supplementary data at *IJE* online) and then code this in Structured Query Language (Supplementary File 4, available as Supplementary data at *IJE* online). Coded fields are extracted from EHR systems, curated to research phenotypes of interest and developed both incrementally and on demand. Search terms are used, including READ2 diagnostic codes, prescribing data, laboratory test results and clinical measurements and processes.

Data concordance was checked between volunteer questionnaires and their EHR, with >99% concordance for gender and year of birth. Almost all cases of data discordance were due to technical errors with questionnaire optical character recognition or user data completion, and were resolved with manual checking. Data outside clinically plausible ranges, or with clear data entry errors, are removed. A detailed description of our data processing is in Supplementary File 3, available as Supplementary data at *IJE* online. Missing data exist, but at relatively low frequency in routinely collected and incentivized clinical measures, e.g. smoking status is recorded in the EHR of 88% of volunteers in the 5 years preceding the most recent data linkage. Repeated measures of routinely collected data and cross-validation across information sources can mitigate the impact of missing data where it exists, as can statistical techniques, such as sensitivity analysis and multiple imputation.^17^

- **NHS local secondary care health record data linkage.** Linkage to Barts Health NHS Trust data provides secondary care data for all ELGH volunteers who have attended this hospital system (24 852 volunteers at the latest linkage). Available data include clinician-coded SNOMED-CT acute and chronic problem lists, laboratory and imaging results. OPCS-4 (Office for Population, Censuses and Surveys) and ICD-10 (World Health Organization International Classification of Diseases and Related Health Problems) codes are available for every finished episode of care. For example, maternity data linkage within Barts Health identified 4172 female ELGH volunteers with maternity records available for one or more pregnancies. Linkage to other local hospitals (including those providing mental and community health care) is planned in 2019/2020.
- **Planned linkage to national health record and other datasets.** ELGH will link to further datasets in 2019/2020, including national NHS Hospital Episode Statistics (HES) and NHS Mortality Data,^18^^,^^19^ to include admissions and discharge, diagnosis and operation codes, maternity, psychiatric and critical care from 1997, and accident and emergency data, ICD-10 and OPCS-4 codes from 2008. NHS mortality data provide data on cause of death. Other planned data linkages to national registries include the National Cancer Registration and Analysis Service and National Cardiovascular Outcomes Research.
- **Genomics.** DNA is extracted from the Oragene (DNA Genotek) saliva system and stored from all Stage 1 volunteers. To date, 20-40X depth exome sequencing has been performed (*n* = 3781) or is in progress (*n* = 1492) on volunteers reporting parental relatedness.

By late 2019 (funding secured), 50 000 samples from stage 1 volunteers will be genotyped on the Illumina Infinium Global Screening Array v3.0 (with an additional 46 662 Multi-Disease variants).^20^ Array content includes rare disease-associated mutations (e.g. all pathogenic and likely pathogenic variants in ClinVar), pharmacogenetic associations and genome-wide coverage for association studies (based on the 26 populations present in Phase III of 1000 Genomes Project, optimized for imputation accuracy), polygenic risk score and Mendelian randomization studies.

In 2019/2020, if support is secured from an evolving Life Sciences Industry Consortium or elsewhere, high-depth exome sequencing will be performed on up to 50 000 volunteer samples. The intention is for genotyping and high-depth exome sequencing to be performed on up to 100 000 volunteer samples by 2023.

- **Samples for other –omics.** Core study samples are taken from all volunteers recalled in stage 2 studies, including a blood cell pellet (for repeat DNA analyses), plasma aliquots and blood cell RNA preservation (Paxgene), for studies including methylation assays, transcriptomics, proteomics, lipidomics and metabolomics.

## What has it found? Key findings and publications

ELGH is a new resource that continues to grow in size and content and, to date, has been used for three main areas of work, as follows.

### Characterization of common phenotypes

Using Type 2 diabetes (T2D) as an exemplar, we show the ability for detailed phenotypic characterization of ELGH volunteers using EHRs (Table 3). Of 21 514 volunteers in ELGH with available linked EHR data, 4769 (22%) have a diagnosis of T2D in their primary care record. Basic sociodemographic data (age, gender, ethnicity) of volunteers were recorded in 100%, and smoking status had been obtained within 2 years of the most recent data linkage in 94%. In over 97% of volunteers with T2D, body mass index, markers of glucose control (HbA1c) and serum cholesterol were measured and available in the 2 years preceding ELGH participation. Hypertension, ischaemic heart disease and chronic kidney disease were observed in 47%, 15% and 11%, respectively, of the 4769, and erectile dysfunction was present in 26% of men. Retinal complications of T2D are recorded and graded, with 82% of volunteers having undergone screening within the past 2 years. Prescribing data show recent insulin prescriptions in 16%, and the use of single or multiple non-insulin agents, as well as use of cardiovascular drugs (e.g. lipid-lowering therapy). These data show the potential to perform cross-sectional analyses in ELGH from EHR data.

**Table 3.** Example of a specific disease phenotype: characteristics of ELGH volunteers with type 2 diabetes. Data are presented in summary and descriptive formats as indicated. Missing data are estimated where available, e.g. for clinical care processes and measurements, but not diagnostic coding where the absence of a code is taken to indicate the absence of a diagnosis

Volunteers with type 2 diabetes = 4769 (22%)				Missing data
Sociodemographic data	Age	Mean years (sd)	46 (11)	0%
	Sex	Male, *n* (%)	2445 (51)	
		Female, *n* (%)	2324 (49)	
	Ethnicity	British Bangladeshi and Bangladeshi, *n* (%)	3860 (81)	
		British Pakistani and Pakistani, *n* (%)	822 (17)	
		Other, *n* (%)	87 (2)	
	Index of Multiple Deprivation (2015)	Quintile 1 (most deprived), *n* (%)	12551 (58)	0%
		Quintile 2, *n* (%)	8400 (39)	
		Quintile 3, *n* (%)	411 (2)	
		Quintile 4, *n* (%)	131 (1)	
		Quintile 5 (least deprived), *n* (%)	11 (0)	
	Smoking status (recorded in the past 2 years)	Data available, *n*	4712	6%
		Never smoked, *n* (%)	3348 (71)	
		Ex-smoker, *n* (%)	810 (17)	
		Current smoker, *n* (%)	554 (12)	
		Coding uninformative, *n* (%)	57 (1)	
	Country of birth	Data available, *n*	2585	46%
		Born in Bangladesh, *n* (%)	2130 (82)	
		Born in Pakistan, *n* (%)	347 (13)	
		Born in England, *n* (%)	54 (2)	
		Born elsewhere, *n* (%)	54 (2)	
Historic T2D data	Age at T2D onset	Data available, *n*	4769	0%
		Mean years (sd)	46 (11)	
	Duration of T2D	Data available, *n*	4769	
		Years (range)	7 (0–51)	
	Diabetes risk state before T2D	Pre-diabetes, *n* (%)	1241 (26)	NA
		Gestational diabetes (females), *n* (%)	370 (16)	
	Body mass index (BMI) at T2D diagnosis	Data available, *n*	3507	5%
		Mean kg/m^2^ (sd)	28.8 (4.9)	
	HbA1c at T2D diagnosis	Data available, *n*	3176	33%
		Mean HbA1c mmol/mol (sd)	61.9 (18.7)	
	Total cholesterol at T2D diagnosis	Data available, *n*	3427	28%
		Mean total cholesterol, mmol/l	5.0 (1.2)	
Current T2D data (recorded within the past 2 years)	Body mass index	Data available, *n*	4630	3%
		Mean kg/m^2^ (sd)	27.9 (4.8)	
	HbA1c	Data available, n	4656	2%
		Mean mmol/mol (sd)	59.2 (15.6)	
	Total cholesterol	Data available, *n*	4640	3%
		Mean total cholesterol, mmol/l	3.8 (1.0)	
	Retinal screening	Data available	4769	NA
		Not in the screening programme, *n* (%)	241 (5)	
		In the screening programme, *n* (%)	4528 (95)	
		Screened in past 2 years, *n* (%)	3926 (82)	
Diabetes complications and multimorbidity	Other diagnoses	Hypertension, *n* (%)	2245 (47)	NA
		Chronic kidney disease, *n* (%)	516 (11)	
		Neuropathy, *n* (%)	164 (3)	
		Ischaemic heart disease, *n* (%)	719 (15)	
		Peripheral vascular disease, *n* (%)	174 (4)	
		Erectile dysfunction (males), *n* (%)	1310 (27)	
		Stroke, *n* (%)	180 (4)	
		Atrial fibrillation, *n* (%)	54 (1)	
		Heart failure, *n* (%)	126 (3)	
	Number of cardiovascular multimorbidities in the presence of type 2 diabetes	One or more conditions, *n* (%)	3804 (80)	
		Two or more conditions, *n* (%)	1291 (27)	
		Three or more conditions, *n* (%)	468 (10)	
		Four or moreconditions, *n* (%)	198 (4)	
		Five or more conditions, *n* (%)	85 (2)	
Drug prescribing	Insulin	Prescribed in the past 12 months, *n* (%)	756 (16)	NA
		Mean years on insulin, (sd)	8.8 (5)	
	Non-insulin diabetes therapies	Metformin prescribed in the past 12 months, *n* (%)	3695 (77)	
		Sulphonylurea prescribed in past 12 months, *n* (%)	1321 (28)	
	Prescribing regimens	Prescribed no non-insulin diabetes therapies, *n* (%)	845 (18)	
		Prescribed one non-insulin diabetes therapy, *n* (%)	2057 (43)	
		Prescribed two non-insulin diabetes therapies, *n* (%)	1127 (24)	
		Prescribed three or more non-insulin diabetes therapies, *n* (%)	740 (16)	
	Lipid-lowering treatment	Prescribed in the past 12 months, *n* (%)	4256 (89)	

NA, not available; sd, standard deviation.

EHR data also give the potential to study longitudinal phenotypic traits, retrospectively and prospectively. Median duration of T2D in ELGH volunteers was 7 years (range 0–51 years). For all volunteers with T2D, year of onset was recorded, and prescribing data and clinical measurements (including body mass index, HbA1c and cholesterol) at the time of diagnosis (+/- 6 months) were available for nearly two-thirds of volunteers. Before T2D onset, 26% (993) had had a diagnosis of pre-diabetes and 16% (370) of women had had a diagnosis of gestational diabetes, allowing study of progression from at-risk to disease states.

Multimorbidity is an increasing problem in ageing populations with high rates of chronic long-term disease; in the ELGH population we identified that of the 4769 ELGH volunteers with T2D, 80% had at least one, and 27% had two or more cardiovascular multimorbidities (Table 3).

### Rare allele frequency gene variants occurring as homozygotes, including predicted loss-of-function knockouts

**Figure 3.**
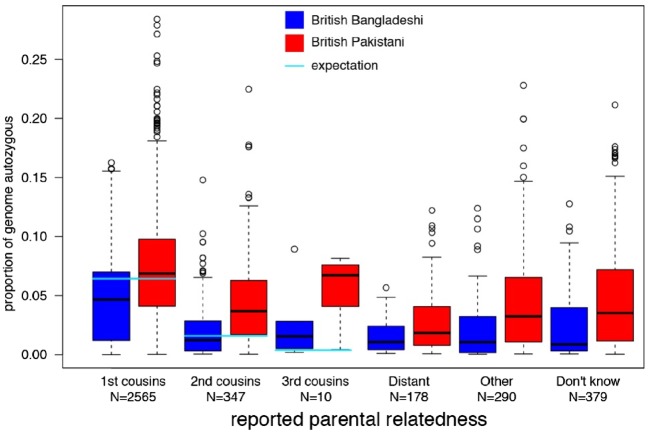
Distribution of levels of autozygosity as a fraction of the genome in ELGH volunteers, split according to self-reported parental relatedness and ethnicity (Tukey box plot showing median, lower and upper quartiles, quartiles +/- 1.5x interquartile range, and outliers).

All ELGH volunteers self-reporting parental relatedness (19%) have been selected for exome sequencing. Genomic autozygosity (homozygous regions of the genome identical by descent from a recent common ancestor) means that rare allele frequency (minor allele frequency <0.5%) variants normally only seen as heterozygotes are enriched for homozygote genotypes. ELGH expands existing, smaller studies of autozygosity to investigate the health and population effects of such variants, with a focus on loss of function variants.^11^^,^^21^^,^^22^ The accuracy of self-reported parental relatedness to actual autozygosity measured at the DNA level by exome sequencing (Figure 3) is a modest predictor of actual autozygosity, e.g. we find 8.2% of individuals who declare that their parents are not related in fact have >2.5% genomic autozygosity. For British Bangladeshi volunteers, mean autozygosity is slightly lower than expected given the reported parental relationship (possibly due to confusion over the meaning of e.g. ‘first cousin’ versus ‘second cousin’), whereas for British Pakistani volunteers, mean autozygosity is slightly higher than expected (possibly due to historical parental relatedness). With an ELGH sample size of 100 000 we estimate we will identify rare variant-predicted loss-of-function homozygotes in >5000 human genes. ELGH plans to work with other studies on an international human knockout variant browser.

### Recall by genotype (and/or phenotype) studies

RbG studies, applied to population cohorts with genomic data, are of increasing research interest^23^ and use the random allocation of alleles at conception (Mendelian randomization) to aid causal inference in population studies, reduce biases seen with observational studies and develop functional studies. RbG studies can target specific single variants (or an allelic series for a gene) and polygenic variants (e.g. extremes of polygenic risk scores).

ELGH is undertaking RbG studies in Stage 2 using bespoke clinical phenotyping tailored to the genotype or phenotype of interest. To date, three research consortia have undertaken ELGH RbG studies, one recalling volunteers with loss-of-function gene variants relevant to immune phenotypes, another phenotyping individuals with rare variants in genes implicated in T2D and obesity and a third involving an industrial partnership to aid therapeutic development for a rare autosomal recessive metabolic disorder.^24^ Successful recall completion rates to these RbG studies are between 30% and 40%.

## What are the main strengths and weaknesses?

ELGH has multiple strengths as a large, population-based study, and its novel, pragmatic design offers opportunities to combine genomic investigation with longitudinal and cross-sectional description of health and disease as determined from EHR data.^25^ ELGH reaches a British Bangladeshi and British Pakistani population with a high burden of disease, generalizable to a wider global population and building on existing genetic studies that have been criticized for focusing on White populations and substantially under-recruiting from minority ethnic groups.^26^ High rates of autozygosity in ELGH volunteers lead to homozygous genotypes at variants with rare allele frequencies that will aid gene discovery, and RbG studies will generate novel translational impact.^11^^,^^24^ Future studies on autozygosity will inform novel population level insights into the impact of genetic variation on health. The ability to invite all volunteers to Stage 2 studies offers the possibility to develop subcohorts and trials within cohorts in the future.

Our community-based recruitment approach offers broad reach into the target population. However, to date, ELGH has modestly over-recruited British Bangladeshi versus British Pakistani volunteers. To support increased recruitment of British Pakistani volunteers, recruitment is expanding into outer London boroughs and a new Bradford Genes & Health.

The use of real-world EHR data is both a strength and weakness of ELGH. Strengths include the ability to obtain longitudinal data available on multiple diseases and disease risks via primary care, in large numbers of volunteers in a feasible and cost-effective manner. Data linkage is not yet complete, but will improve in 2019 with improved infrastructure and linkage to national registries and databases. Weaknesses are that EHR data may be inferior to observational epidemiological studies in ascertaining some phenotypes, e.g. recent diseases of minor severity (which do not necessarily require health care access) or subclinical disease. Additionally, although outcomes can be studied relatively well, EHR data have limited opportunity to study certain exposures, e.g. health behaviours, physical activity, diet and some other environmental influences.

## Can I get hold of the data? Where can I find out more?

ELGH offers an open access resource to international, academic and industrial researchers to drive high-impact, world-class science. Data access is managed at several levels, as follows.

- Level 1. Fully open data: summary data are distributed via our website, e.g. genotype counts and annotation of knockout variants from exome sequencing, and prevalence of phenotype and traits data.
- Level 2. Genotype data (SNP chip genotyping, or high-throughput sequencing) are (or will be) available under data access agreements granted by the independent Wellcome Sanger Institute Data Access Committee. Individual sequencing (e.g. cram) and genotype files (e.g. vcf) are available within 6 months on the European Genome-phenome Archive^27^ (EGA).
- Level 3. Individual-level phenotype data are held in an ISO27001 compliant data safe haven environment under data access agreement, currently hosted by the UK Secure e-Research Platform.^28^^,^^29^ The data safe haven contains the latest genetic data linked to the questionnaire and health record phenotypes, and data export is tightly controlled. This ‘bring researchers to the data’ model allows us to share regular data updates, maintain complex data linkages and avoid large file data transfers. This model provides robust reassurance to volunteers that their health data will be carefully looked after, with maximum security against data breaches.

External researchers can to apply to undertake research with ELGH via a formal application process(details are available on the website), and most will be required to have their own research ethics approval to work with ELGH. Applications are assessed by both the executive board and community advisory group, according to community prioritization, acceptability and scientific merit.

**Profile in a nutshell**

- East London Genes & Health (ELGH) is a large-scale, community genomics and health study (to date 38 899 volunteers; target 100 000 volunteers).
- ELGH was set up in 2015 to gain deeper understanding of health and disease, and underlying genetic influences, in British Bangladeshi and British Pakistani people living in east London.
- ELGH prioritizes studies in areas important to, and identified by, the community it represents. Current priorities include cardiometabolic diseases (high prevalence of early onset) and mental illness. However, studies in any scientific area are possible, subject to community advisory group and ethical approval.
- ELGH combines health data science [using linked UK National Health Service (NHS) electronic health record data] with exome sequencing and SNP array genotyping to elucidate the genetic influence on health and disease, including the contribution from high rates of parental relatedness to rare genetic variation and homozygosity (autozygosity), in two understudied ethnic groups. Linkage to longitudinal health record data enables both retrospective and prospective analyses.
- Through Stage 2 studies, ELGH offers researchers the opportunity to undertake recall-by-genotype and/or recall-by-phenotype studies on volunteers. Subcohort studies, trials within cohort and other study designs are possible.
- ELGH is a fully collaborative, open access resource, open to academic and life sciences industry scientific research partners.

## Funding

We acknowledge funding from the Wellcome Trust (102627, 210561), the Medical Research Council (M009017), Higher Education Funding Council for England Catalyst, Barts Charity (845/1796), Health Data Research UK and the NHS National Institute for Health Research Clinical Research Network.

## Supplementary Material

dyz174_Supplementary_Data

## References

[1] Popejoy AB, Fullerton SM. Genomics is failing on diversity. Nature 2016;538:161–64. 10.1038/538161a PMC5089703 27734877

[2] Office For National Statistics. *2011* Census: Ethnic Group, Local Authorities in the United Kingdom. London: ONS, 2013.

[3] Barnett AH, Dixon AN, Bellary S et al Type 2 diabetes and cardiovascular risk in the UK South Asian community. Diabetologia 2006;49:2234–46. 16847701 10.1007/s00125-006-0325-1

[4] Sattar N, Gill J. Type 2 diabetes in migrant south Asians: mechanisms, mitigation, and management. Lancet Diabetes Endocrinol 2015;3:1004–16. 26489808 10.1016/S2213-8587(15)00326-5

[5] Goodyear M. *Public Health Profile of North East London for NE London Sustainability and Transformation Plan* 2016 http://archive.eastlondonhcp.nhs.uk/wp-content/uploads/2017/06/NEL-STP-JSNA-2016.pdf (6 September 2018, date last accessed).

[6] Mathur R, Noble D, Smith D, Greenhalgh T, Robson J. Quantifying the risk of type 2 diabetes in East London using the QDScore: a cross-sectional analysis. Br J Gen Pract 2012;62:e663–70. 23265225 10.3399/bjgp12X656793 PMC3459773

[7] Alazawi W, Mathur R, Abeysekera K et al Ethnicity and the diagnosis gap in liver disease: a population-based study. Br J Gen Pract 2014;64:e694–702. 25348993 10.3399/bjgp14X682273 PMC4220229

[8] George J, Mathur R, Shah AD et al Ethnicity and the first diagnosis of a wide range of cardiovascular diseases: Associations in a linked electronic health record cohort of 1 million patients. PLoS One 2017;12:e0178945. 28598987 10.1371/journal.pone.0178945 PMC5466321

[9] Khera AV, Chaffin M, Aragam KG et al Genome-wide polygenic scores for common diseases identify individuals with risk equivalent to monogenic mutations. Nat Genet 2018;50:1219–24. 30104762 10.1038/s41588-018-0183-z PMC6128408

[10] Eric Vallabh M, Karczewski KJ, Martin HC et al Evaluating Potential Drug Targets Through Human Loss-of-function Genetic Variation. https://www.biorxiv.org/content/10.1101/530881v2 (29 January 2019, date last accessed).

[11] Narasimhan VM, Hunt KA, Mason D et al Health and population effects of rare gene knockouts in adult humans with related parents. Science 2016;352:474–77. 10.1126/science.aac8624 PMC4985238 26940866

[12] Joshi PK, Esko T, Mattsson H et al Directional dominance on stature and cognition in diverse human populations. Nature 2015;523:459–62. 26131930 10.1038/nature14618 PMC4516141

[13] Centre of the Cell. Centre of the Cell. https://www.centreofthecell.org/.

[14] Dreyer G, Hull S, Mathur R, Chesser A, Yaqoob MM. Progression of chronic kidney disease in a multi-ethnic community cohort of patients with diabetes mellitus. Diabet Med 2013;30:956–63. 23600455 10.1111/dme.12197

[15] Hull S, Chowdhury TA, Mathur R, Robson J. Improving outcomes for patients with type 2 diabetes using general practice networks: a quality improvement project in east London. BMJ Qual Saf 2014;23:171–76. 10.1136/bmjqs-2013-002008 24003237

[16] Robson J, Dostal I, Madurasinghe V et al NHS Health Check comorbidity and management: an observational matched study in primary care. Br J Gen Pract 2017;67:e86–93. 27993901 10.3399/bjgp16X688837 PMC5308122

[17] Farmer R, Mathur R, Bhaskaran K, Eastwood SV, Chaturvedi N, Smeeth L. Promises and pitfalls of electronic health record analysis. Diabetologia 2018;61:1241–48. 10.1007/s00125-017-4518-6 PMC6447497 29247363

[18] NHS Digital. Linked HES ONS Mortality Data, 2015. https://digital.nhs.uk/binaries/content/assets/legacy/pdf/r/q/hes-ons_linked_mortality_data_guide.pdf or https://digital.nhs.uk/data-and-information/data-tools-and-services/data-services/linked-hes-ons-mortality-data (6 August 2019, date last accessed).

[19] NHS Digital. Hospital Episode Statistics. https://digital.nhs.uk/data-and-information/data-tools-and-services/data-services/hospital-episode-statistics (6 August 2019, date last accessed).

[20] Illumina. GSA Array Datasheet, 2018. https://www.illumina.com/content/dam/illumina-marketing/documents/products/datasheets/infinium-commercial-gsa-data-sheet-370-2016-016.pdf (6 August 2019, date last accessed).

[21] Narasimhan VM, Rahbari R, Scally A et al Estimating the human mutation rate from autozygous segments reveals population differences in human mutational processes. Nat Commun 2017;8:303. 28827725 10.1038/s41467-017-00323-y PMC5566399

[22] Saleheen D, Natarajan P, Armean IM et al Human knockouts and phenotypic analysis in a cohort with a high rate of consanguinity. Nature 2017;544:235–39. 28406212 10.1038/nature22034 PMC5600291

[23] Corbin LJ, Tan VY, Hughes DA et al Formalising recall by genotype as an efficient approach to detailed phenotyping and causal inference. Nat Commun 2018;9:711. 29459775 10.1038/s41467-018-03109-y PMC5818506

[24] McGregor TL, Hunt KA, Nioi P et al *Deep Phenotyping of a Healthy Human HAO1 Knockout Informs Therapeutic Development for Primary Hyperoxaluria Type 1* https://www.biorxiv.org/content/10.1101/524256v1 (28 January 2019, date last accessed).

[25] Prados-Torres A, Poblador-Plou B, Gimeno-Miguel A et al Cohort Profile: The epidemiology of chronic diseases and multimorbidity. The EpiChron cohort study. Int J Epidemiol 2018;47:382–84. 29346556 10.1093/ije/dyx259 PMC5913592

[26] Kaiser J, Gibbons A. Biology in the bank. Science 2019;363:18–20. 30606827 10.1126/science.363.6422.18

[27] European Bioinformatics Institute. European Genome-Phenome Archive. https://www.ebi.ac.uk/ega (18 September 2018, date last accessed)

[28] Jones KH, Ford DV, Ellwood-Thompson S, Lyons RA. The UK secure eResearch platform for public health research: a case study. Lancet 2016;388:S62.

[29] Ford DV, Jones KH, Verplancke J-P et al The SAIL Databank: building a national architecture for e-health research and evaluation. BMC Health Serv Res 2009;9:157. 19732426 10.1186/1472-6963-9-157 PMC2744675

