# Supplementary figures and images for: Cohort Profile: East London Genes & Health (ELGH), a community-based population genomics and health study in British Bangladeshi and British Pakistani people
Source: Int J Epidemiol. 2019 Aug 28;49(1):20–21i. doi: 10.1093/ije/dyz174 (PMC7124496; doi:10.1093/ije/dyz174)

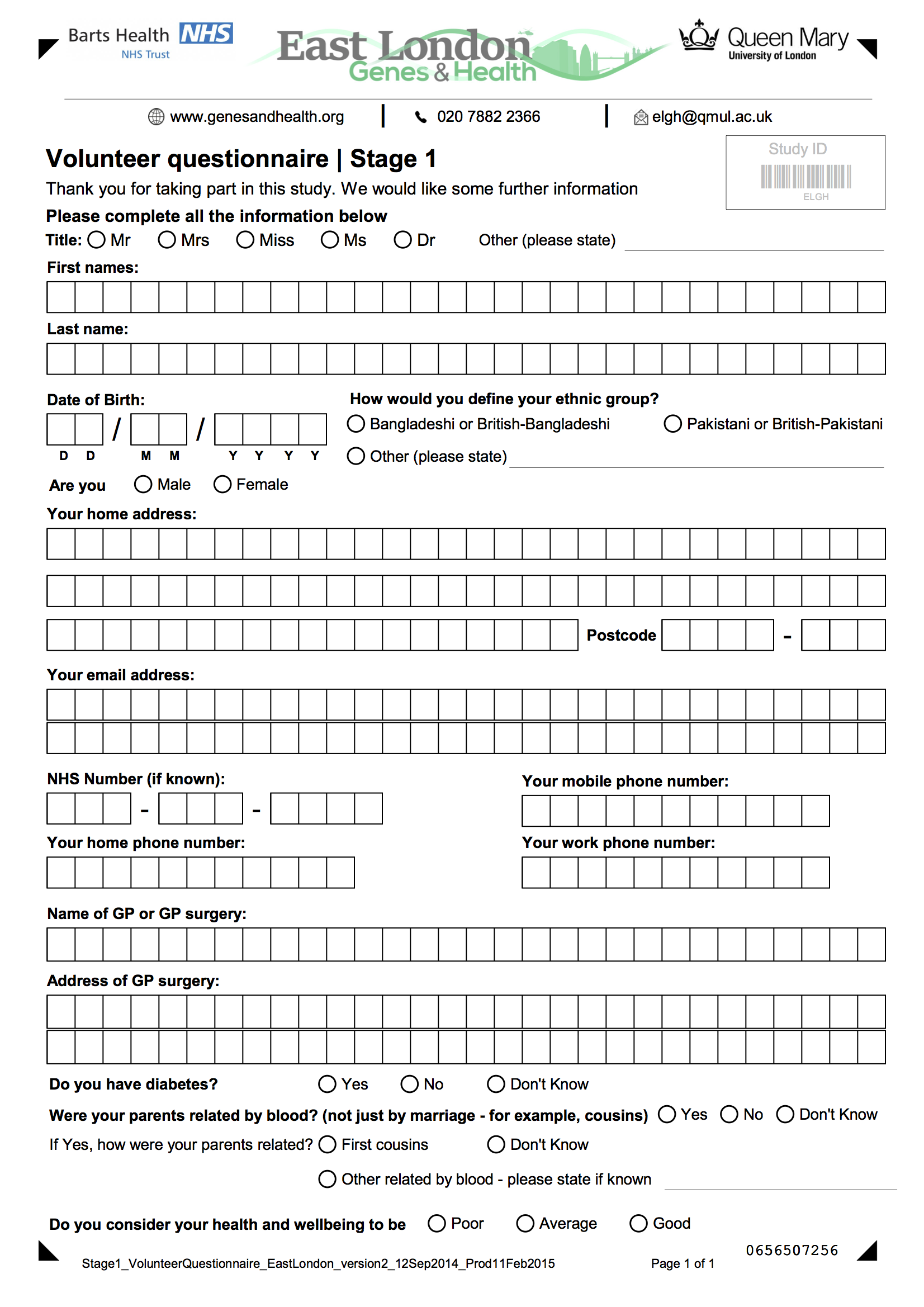

Supplement: dyz174_Supplementary_Data [file dyz174_supplementary_data.zip › dyz174-suppl_data/ije-2019-02-0223-File008.png]
